# Minor Hospital War News

**Published:** 1914-08-15

**Authors:** 


### Minor Hospital War News.

Sir Robert and Lady Harvey, of Langley Park, Slough, have offered their house to the St. John Ambulance as a hospital or convalescent home for officers.

Colonel Burn, M.P., Sir Francis Layland-Barrett, and Mrs. Dundee Hooper have offered their houses at Torquay for similar purposes.

Lord Harrington has offered to place Harrington House, Kensington Palace Gardens, W., at the disposal of the Committee of the City of London Branch of the British Red Cross Society.

The Committee of the Providence Free Hospital, St. Helens, Lancashire, have placed at the disposal of the authorities all their available accommodation for the treatment of sick and wounded soldiers and sailors.

Harestone Hall, Caterham, is offered for the purposes of a temporary hospital for officers.

It is interesting to note that twenty-five beds have been offered by the Committee of Management of the Italian Hospital, Queen Square, W.C.

### Gifts and the War.

The Empress Eugenie has sent £200 to the funds of the British Red Cross Society. The total amount of the special subscriptions received since Saturday exceeds £8,000.

